# Medical Certificates and School Attendance

**Published:** 1905-06-10

**Authors:** 


### Medical Certificates and School Attendance.

The Medico-Political Committee of the British Medical Association, guided doubtless by expert advice, has formulated the legal position with respect to medical certificates of unfitness of children to attend school. The demand for these certificates is an annoying experience to many practitioners in the poorer districts, and also to the out-patient staffs of hospitals. Parents expect to obtain them without a fee and are able to urge their claim by the fact that the absence of the certificate will almost certainly lead to the issue of a summons. These facts have led to a demand that the local educational authority should either pay for these certificates or should appoint a medical officer or officers charged with the duty of determining whether or not in each individual case the plea of illness is a valid one. Possibly one or other of these plans is the reasonable and fair method of settling the difficulty, but it appears certain that the legal position does not demand, perhaps does not even authorise, such action on the part of the local educational authority. According to expert construction of the statute, it lies with the parent whose child is withheld from school to prove the existence of a "reasonable excuse," and on him, therefore, in a case of alleged illness, falls the onus of proof. Hence, technically at least, the provision of a certificate by a medical practitioner is an act performed on behalf of the parent, and does not in any legal sense establish a relationship between the practitioner and the educational authority. Similarly the dispute whether the certificate should or should not contain a statement of the nature of the illness finds its conclusion in the decision of the magistrate with whom it rests to say whether the certificate is or is not adequate. Indeed, it is open to the magistrate to decline to accept any certificate and to demand that medical evidence, if it exists, shall be given in the witness-box on oath and subject to the discipline of cross-examination.

No doubt all this, in view of the authority by which it is issued, is, in the forensic sense of the term, "good law," but it would have been more satisfactory had the medico-political committee afforded some help in the practical solution of the problem as it actually exists in the work and experience of many practitioners. Presumably, if the letter of the law is to be strictly followed, it is open to any parent who withholds his child from school to decline to take any steps to substantiate the plea of illness until he is actually summoned before a magistrate. He may consider he has "reasonable excuse" for keeping the child at home and may accept the risk of a summons by the educational authority feeling satisfied that he can convince the magistrate of the soundness of his position. In doing this he may summon as a witness a medical practitioner who in turn may, before he gives his evidence, advance a claim for the payment of his fee. And in such a position the medical witness may under the pressure of the Court be compelled to reveal the nature of the child's illness. In the end, it is readily conceivable that the parent might establish his position, and the action of the educational authority be shown to have no valid basis. Were such a policy as this widely adopted both parents and medical practitioners would no doubt be put to much trouble, but the educational authorities would at least be equal sufferers. What actually happens, of course, is that the educational authority scrutinises the certificates and other evidence in each case, and so informally determines whether the excuse for non-attendance is or is not genuine. Therefore, in substance, if not in form, the medical certificate is a contribution to the smooth and economical working of the educational machinery. Hence there is an obvious moral claim for its recognition by the educational exchequer. If the matter is to be argued as a technical right, the question of "proof" lies with the magistrate and not with the educational authority. But in this, as in many other practical problems, convenience and efficiency are best secured by the application of common sense rather than by a rigid interpretation of statutory claims. It is to be hoped, therefore, that the Medico-Political Committee will not content themselves with a mere statement of the formal legal position, but will address their energies to the arrangement of a policy which will secure justice to all concerned. The question is one for practical measures, not for forensic discussion.

